# Evaluation of microRNA-10b prognostic significance in a prospective cohort of breast cancer patients

**DOI:** 10.1186/1476-4598-13-142

**Published:** 2014-06-04

**Authors:** Paola Parrella, Raffaela Barbano, Barbara Pasculli, Andrea Fontana, Massimiliano Copetti, Vanna Maria Valori, Maria Luana Poeta, Giuseppe Perrone, Daniela Righi, Marina Castelvetere, Michelina Coco, Teresa Balsamo, Maria Morritti, Fabio Pellegrini, Andrea Onetti-Muda, Evaristo Maiello, Roberto Murgo, Vito Michele Fazio

**Affiliations:** 1 Laboratory of Oncology, IRCCS Casa Sollievo della Sofferenza, Viale Padre Pio, 71013 San Giovanni Rotondo, (FG), Italy; 2 Department of Biosciences, Biotechnology and Biopharmaceutics, University of Bari “Aldo Moro”, Bari, Italy; 3 Unit of Biostatistics, IRCCS Casa Sollievo della Sofferenza, San Giovanni Rotondo, Italy; 4 Department of Oncology, IRCCS Casa Sollievo della Sofferenza, San Giovanni Rotondo, Italy; 5 Department of Pathology, University Campus Bio-Medico, Rome, Italy; 6 Department of Pathology, IRCCS Casa Sollievo della Sofferenza, San Giovanni Rotondo, Italy; 7 Unit of Biostatistics, DCPE Fondazione Mario Negri Sud, Santa Maria Imbaro, (CH), Italy; 8 Breast Unit, IRCCS Casa Sollievo della Sofferenza, San Giovanni Rotondo, Italy; 9 CIR Laboratory for Molecular Medicine and Biotechnology, University Campus Biomedico, Rome, Italy

**Keywords:** Breast cancer, microRNA, Metastasis, RT-qPCR

## Abstract

**Background:**

MicroRNA-10b (miR-10b) has a prominent role in regulating tumor invasion and metastasis by targeting the HOXD10 transcriptional repressor and has been found up-regulated in several tumor types.

**Methods:**

We evaluated the expression of miR-10b in paired tumor and normal specimens obtained from a prospective cohort of breast cancer patients with at least 36 months follow-up enrolled according to the REMARK guidelines (n = 150). RNA quality was measured and only samples with RNA Integrity Number (RIN) ≥7.0 were analyzed.

**Results:**

The relative expression of miR-10b in tumor as compared to its normal counterpart (RER) was determined by RT-qPCR. miR-10b RERs were higher in the subgroup of patients with synchronous metastases (n = 11, Median 0.25; IQR 0.11-1.02) as compared with patients without metastases (n = 90, Median 0.09; IQR 0.04-0.29) (*p* = 0.028). In the subgroup of patients without synchronous metastases (n = 90), higher miR-10b RERs were associated with increased risk of disease progression and death in both univariable (HR 1.16, *p* = 0.021 and HR 1.20, *p* = 0.015 respectively for 0.10 unitary increase of miR-10b RERs levels) and multivariable (HR1.30, *p* < 0.001, and HR 1.31, *p* = 0.003 respectively for 0.10 unitary increase of miR-10b RERs levels) Cox regression models. The addition of miR-10b RERs to the Nottingham Prognostic Index (NPI) provided an improvement in discrimination power and risk reclassification abilities for the clinical outcomes at 36 months. Survival C-indices significantly increased from 0.849 to 0.889 (*p* = 0.009) for OS and from 0.735 to 0.767 (*p* = 0.050) for DFS.

**Conclusions:**

Our results provide evidences that the addition of miR-10b RERs to the prognostic factors used in clinical routine could improve the prediction abilities for both overall mortality and disease progression in breast cancer patients.

## Background

In recent years mortality from breast cancer has declined in western countries likely as a result of more widespread screening resulting in earlier detection, as well as advances in the adjuvant treatment [1]. Several prognostic factors are currently used in routine practice to select patients most likely to recur without adjuvant therapy and therefore that potentially benefit from therapy. However, even patients with better prognosis may develop metastases and die for the disease [2]. Recent studies have shown that the metastatic capability of cancer is conferred by molecular changes arising relatively early in tumorigenesis and metastatic dissemination may occur continually throughout the course of primary tumor development [3].

MicroRNAs (miRNAs) are small cellular RNAs modulating gene expression at post-transcriptional level [4]. miR-10b was initially found highly expressed in metastatic breast cancer cell lines, able to generate metastases when growing as primary tumor in mice [5]. Moreover, miR-10b silencing by antagomirs markedly suppresses metastases formation in the 4 T1 mouse model although has no effects on tumor growth [6]. The mechanisms by which miR-10b is involved in metastatic processes have been extensively studied in breast cancer cell lines as well as in cells derived by other tumor types [7]. miR-10b has a prominent role in regulating tumor invasion and metastasis by targeting the HOXD10, a transcriptional repressor involved in cellular migration and extracellular modelling such as RhoC, uPAR, α3-integrin and MT1-MMP [5,7-12].

In their original study, Ma and colleagues [5] evaluated miR-10b expression relative to normal mammary tissue in 23 advanced stage breast cancers, finding higher miR-10b levels in metastatic tumors as compared with non-metastatic cancers. A correlation between elevated miR-10b expression and poor prognosis was recently reported in gastric cancer, renal cancer, colorectal tumors, pancreatic cancer and bladder tumors [11,13-19]. Moreover, higher miR-10b expression levels were recently detected in serum from metastatic breast cancer patients [20].

To further clarify the role of miR-10b as prognostic biomarker in breast cancer, we evaluated the association between miR-10b expression and clinical outcome in a cohort of prospectively collected breast cancer tissues.

## Results

### Patients and treatment

Table 1 summarizes descriptive statistics for the 101 cases selected for our analysis. The median age of the study population is 59 years (range, 36 to 82), median tumor size is 2.5 cm (range, 1.0 to 10.0). Metastases at diagnosis (synchronous metastases) were present in 11 cases whereas, among non-metastatic patients, 34 experienced disease progression and 30 of them developed distant metastases (metachronous metastases).

**Table 1:** Clinicopathological characteristics of the patients cohort (n = 101)

**Characteristics**		**n**	**%**
**Tumor histotype**	Ductal	92	91.1
	Lobular	7	6.9
	Others	2	2.0
**Tumor**	T1c	27	26.7
	T2	45	44.6
	T3	4	4.0
	T4	25	24.7
**Lymph nodes**	N0	34	33.7
	N1	32	31.7
	N2	15	14.8
	N3	20	19.8
**Metastases**	Absent	90	89.1
	Present	11	10.9
**Stage**	I	15	14.8
	II	45	44.6
	III	30	29.7
	IV	11	10.9
**ER status**	Negative	38	37.6
	Positive	63	62.4
**PgR status**	Negative	50	49.5
	Positive	51	50.5
**HER2 amplification**	Negative	66	65.3
	Positive	30	29.7
	Missing	5	5.0
**Receptor Classification**	Receptor positive	63	62.4
	Triple Negative	20	19.8
	Her2/neu amplified	18	17.8
**Grade**	G1	11	10.8
	G2	38	37.7
	G3	40	39.6
	Missing	12	11.9
**First metastatic site**	Bone	19	46.4
	Lung	11	26.8
	Brain	6	14.6
	Liver	2	4.9
	Others	3	7.3
**NPI**	Low Risk	16	18.0
	Intermediate Risk	43	48.3
	High Risk	30	33.7
Adjuvant therapy*	HT + CT	53	58.9
	CT	18	20.0
	Ct + anti-HER2	16	17.8
	HT	2	2.2
	None	1	1.1

*HT, Hormone Therapy; CT, Chemotherapy.

All patients received adequate local treatment (breast conserving surgery or total mastectomy) plus sentinel node biopsy or complete axillary dissection. Post-surgery treatments were performed according to the following guidelines: San Gallen, NCCN and ASCO. Adjuvant therapy in association with postoperative breast irradiation (RT) was performed in 89 patients because one subject refused treatment.

### Evaluation of miR-10b expression in breast tissues by RT-qPCR

miR-10b expression was evaluated in paired normal and tumor tissues obtained from 101 patients. As expected from previous studies [7,21-23] overall miR-10b expression levels (miR-10b/RNU48x1000) were lower in tumor tissues as compared with normal breast with median values of 28.33 (IQR 10.68-62.71) and 254.95 (IQR 110.28-495.09). Thus, to determine tumor specific changes we evaluated for each patient the ratio between the levels of miR-10b expression in cancer specimen to the levels of miR-10b expression in paired normal tissue (RER). RERs ranged from 0.05 to 1.7 with a median value of 0.10 (IQR 0.05-0.32).

### Association of miR-10b RERs with distant metastases

The only significant association with clinicopathological characteristics was found between miR-10b RERs and the presence of distant metastases at diagnosis (Additional file 1: Table S1). miR-10b RERs were significantly higher in the subgroup of patients with metastases (Median 0.25; IQR 0.11-1.02) as compared with patients without metastases (Median 0.09; IQR 0.04-0.29) (*p* = 0.028). No statistically significant difference was found in miR-10b RERs between patients with synchronous (N = 11) and metachronous metastasis (N = 30) (*t*-test p = 0.096).

In the 41 patients with synchronous or metachronous distant metastases, the group of patients with brain metastases (n = 6) had significantly higher miR-10b RERs (Median 0.47; IQR 0.20-1.62) as compared with patients (n = 35) showing metastases in other organ sites (Median 0.10; IQR 0.03-0.61) (*t*-test p = 0.043) (Additional file 1: Table S1).

### HOXD10 protein expression is inversely correlated with miR-10b expression levels in breast tissues

**Figure 1:**
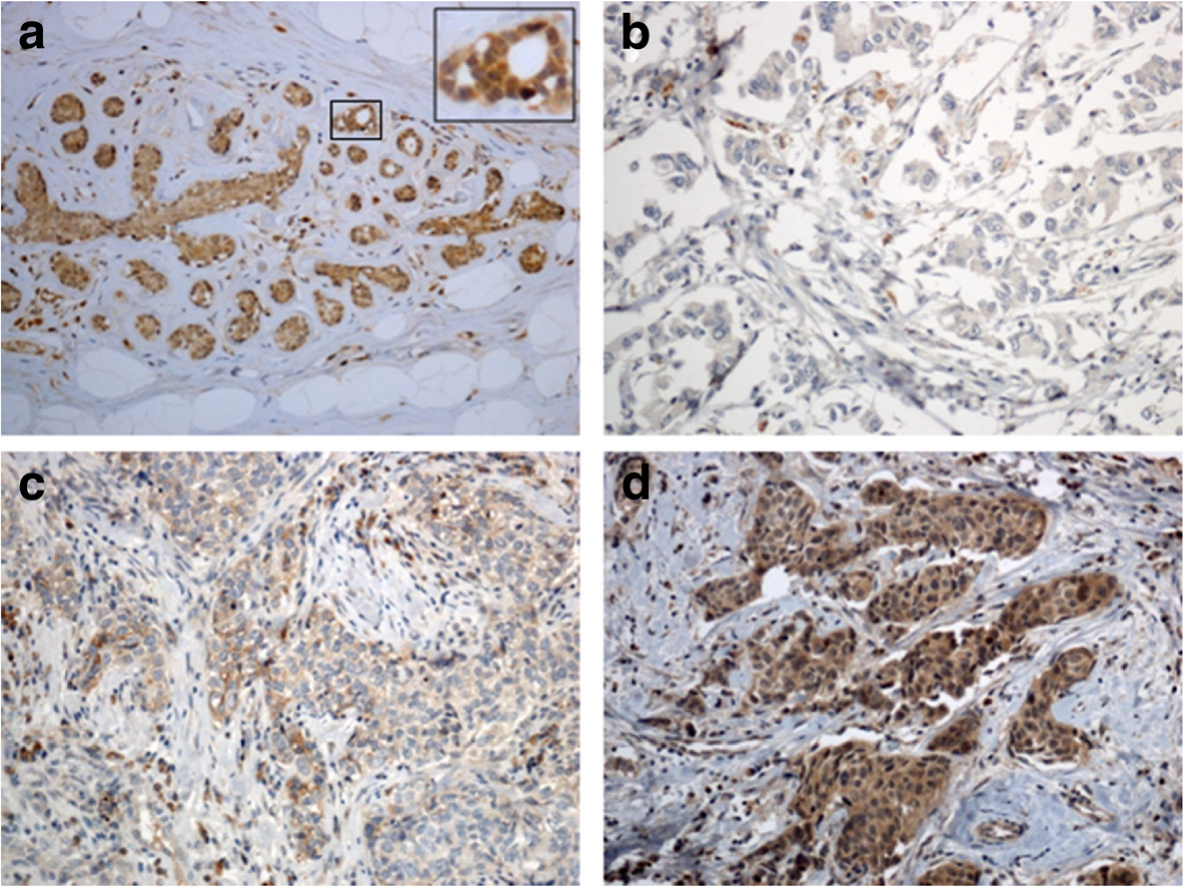
**HOXD10 protein expression by immunohistochemistry. a)** representative image of normal breast epithelium from an healthy individual (HBS1): HOXD10 was constitutively expressed in normal ductal and lobular epithelium and therefore were used as internal positive control. **b)** representative image of breast cancer case BC4 developing distant metastases (brain, bone, liver) during follow-up: miR-10b RERs were 0.78 and HOXD10 protein was expressed in 20% of cancer cells; **c)** and **d)** representative images of BC6 and BC7 non-metastatic breast cancer cases. HOXD10 showed a diffuse staining, miR-10b RERs were 0.01 and 0.42 respectively and percentage of stained cells were 70% and 100% respectively. Original magnification: 100X **a**, **b**, **c**, **d** images; 400X squared area of an image.

We evaluated by IHC the expression of miR-10b target gene HOXD10 in three normal breast tissues from reductive mammoplasty and 10 paired normal and tumor tissues (Additional file 1: Table S1). HOXD10 was constitutively expressed in normal ductal and lobular epithelium (Figure 1a). In tumor tissues HOXD10 was variably expressed with tumors showing a diffuse immunostaining (Figure 1c-d) and tumors with a low percentage of stained cancer cells (Figure 1b). A statistically significant inverse correlation was found among miR-10b expression levels and percentage of HOXD10 expressing cells (Spearman Rho −0.713 *p* < 0.001).

### Association of miR-10b RERs with survival in patients without synchronous metastases

The association with survival was evaluated by using miR-10b RERs values as a continuous variable in the group of patients without metastases at diagnosis (n = 90). In univariable Cox regression model, patients with higher miR-10b RERs showed an increased risk of disease progression (HR1.16, *p* = 0.021), metastases development (HR1.17, *p* = 0.019), and cancer related death (HR1.20; *p* = 0.015) (Table 2). Other factors associated with outcome in univariable analysis are shown in Additional file 1: Table S2. A multivariable Cox regression model adjusted for tumor size, lymph node metastases, Grade, ER and PgR status, and KI67 labeling index (n = 79), confirmed that increased miR-10b RERs were associated with higher risk of disease progression (HR1.30; *p* < 0.001), distant metastases (HR1.34; *p* < 0.001) and worse overall survival (HR1.31; *p* = 0.003) (Table 2). The assumption of proportional hazards was satisfied.

**Table 2:** Proportional hazards Cox regression models evaluating the association between miR-10b RERs and Overall Survival (OS), Disease Free Survival (DFS) and Metastases Free Survival (MFS) in the 90 cases without metastases at diagnosis

**Outcome**	Median follow-up (range)*	**Events/Total**	**Model**	**HR**	**95% CI**	***p***
Disease Free Survival (DFS)	39.88 (21.17 – 58.77)	34/90	Univariable	1.16	1.02-1.31	**0.021**
		31/79	Multivariable	1.30	1.11-1.51	**<0.001**
Metastasis-Free Survival (MFS)	43.57 (25.23 – 59.77)	30/90	Univariable	1.17	1.03-1.34	**0.019**
		27/79	Multivariable	1.34	1.13-1.59	**<0.001**
Overall Survival (OS)	45.78 (32.60 – 60.50)	18/90	Univariable	1.20	1.04-1.38	**0.015**
		16/79	Multivariable	1.31	1.10-1.57	**0.003**

*Abbreviations*: *HR* Hazard Ratio, 95% CI, 95% Confidence Interval.

Multivariable models included: T, N, Grade, ER, PgR, HER2 and KI67.

HR were reported for each unitary increase of 0.1 miR-10b RERs.

### Association of miR-10b RERs with response to adjuvant treatment

The association of miR-10b RERs with response to therapy in terms of DFS and MFS was also evaluated. In univariable Cox regression analysis, a statistically significant association between higher RERs and risk of metastases development was found in the subgroup of patients treated with hormone therapy in association with chemotherapy (HR1.22, *p* = 0.039). A trend toward an association was found for the same subgroup with DFS (HR1.18, *p* = 0.063) (Additional file 1: Table S3).

### Performance of NPI and NPI + miR-10b RERs in predicting short term outcome in the patient population

We evaluated whether the addition of miR-10b RERs to the model with NPI index alone was able to provide improvements in discriminatory power and risk reclassification abilities for the clinical outcomes at 36 months. As shown in Table 3, the survival C-indices significantly increased from 0.849 to 0.889 (*p* = 0.009) for OS and from 0.735 to 0.767 (*p* = 0.050) for DFS with the inclusion of miR-10b RERs, along with a very good calibration (all HL *p*-values were greater than 0.94) for the OS and DFS outcomes, respectively. Furthermore, the addition of miR-10b RERs to the NPI for OS allowed to correctly reclassify 31 out of 89 patients, where 1 of 11 were events (10.4%) and 30 of 70 were non-events (43.4%), providing a cNRI of 0.538 (*p* = 0.061).

**Table 3:** Measures of model performance of Nottingham Prognostic Index (NPI), without and without miR-10b RERs

**A) Overall survival**					
**Model**	**Calibration (*p*-value)**	**Survival C-index (95% CI)**	**Difference in C-index (*p-*value)**	**cNRI (95% CI)**	**cNRI (*p*-value)**
NPI	0.999	0.849 (0.78-0.92)	0.009	0.538	0.061
NPI+miR-10b RERs	0.999	0.889 (0.82-0.96)		Events: 1/11	
				Nonevents: 30/70	
**B) Disease free survival**					
NPI	0.999	0.735 (0.656-0.815)	0.050	0.496	0.015
				Events: 1/25	
				Nonevents: 30/56	
NPI+miR-10b RERs	0.942	0.767 (0.676-0.859)			

The addition of miR-10b RERs to the NPI for DFS allowed to correctly reclassify 29 out of 89 patients where: only 30 of 56 non-events (53.8%) were correctly reclassified while 1 of 25 events (4.2%) was misclassified, providing therefore a cNRI of 0.496 (*p* = 0.015). Therefore, a large proportion of non-events were correctly reclassified when considering both NPI and miR-10b RERs into the prediction models for both clinical outcomes.

## Discussion

MicroRNA-10b was identified as a miRNA highly expressed in metastatic breast cancer cell lines, able to generate metastases when growing as primary mammary tumor in mice [5,6]. Although Gee and colleagues [21] did not find association between miR-10b and outcome in a retrospective breast cancer cohort, an association between elevated miR-10b expression and poor prognosis was reported in several tumor types [11,13-19]. Thus, we took the effort to further evaluate the putative role of miR-10b as prognostic biomarker in breast cancer by analyzing a cohort of prospective collected cases with at least 3 years follow-up from our tumor bank.

To overcome the variability of miR-10b expression in normal breast tissues and tumor samples [7,21-23], we developed a reliable RT-qPCR approach for the detection of changes directly linked to cancer phenotype. Although cancer samples can be enriched of tumor cells by performing laser microdissection, recent studies suggest that tumor microenvironment plays a pivotal role in maintaining malignant phenotypes [24]. Therefore the analysis of whole tumor tissues is likely to be more informative and accurate than the analysis of isolated epithelial component. The goodness of our analytical approach is further sustained by the inverse correlation found in tissues between miR-10b levels determined by RT-qPCR and the expression of miR-10b target HOXD10 by immunostaining. This result is more remarkable considering that miR-10b expression analysis and HOXD10 immunostaining were performed on two independent samples.

We show that although miR-10b is overall down regulated in tumors with a median RER value of 0.10, metastatic breast cancers show significantly higher RERs (median 0.25) than non-metastatic tumors (median 0.09), thus confirming the initial data by Ma and colleagues [5]. Interestingly, a recent study reported increased miR-10b expression in serum obtained from breast cancer patients with higher levels in metastatic tumors as compared with non-metastatic cancers (20). Moreover, Chan and colleagues [25] demonstrated that while miR-10b is down regulated in tumor tissues as compared to normal breast, it shows overexpression in corresponding serum specimens. These data are consistent with our results and might be explained by the existence of a miR-10b over-expressing subpopulation within primary tumor responsible of miR-10b shed in the bloodstream. We can speculate that the more this miR-10b overexpressing subpopulation is represented in primary tumor the higher is the risk for the patient to develop distant metastases.

In our cohort, patients showing higher miR-10b RER were more likely to progress, develop metastases and die for the disease. These associations are independent from the prognostic factors used in routine practice to stratify patients according to their risk to progress. Our results also suggest that high miR-10b RERs might be involved in primary resistance to hormone therapy, although these data are limited by the small sample size of therapies subgroups.

A limitation of our study is the scarce representation in the population of cases classified at low risk by the NPI. This is mainly due to the restrictions for tumor banking which allow only the collection of tumor greater than 1.0 cm in diameter, thus affecting one of the main factors included in the NPI. Nevertheless, we found that the addition of miR-10b RERs to the NPI for the prediction of both overall mortality and disease progression risks in breast cancer patients significantly increased the model’s discriminatory power and the risk reclassifications within 36 months of follow up.

## Conclusions

This study provides evidences that miR-10b expression is associated with clinical outcome in breast cancer patients. If these results will be confirmed on a longer follow-up, miR-10b RERs could be used as biomarkers for a better patient’s risk stratification. Lower miR-10b RERs identify those breast cancer patients who despite having clinical features associated with adverse outcome might not need intensive adjuvant treatment. Moreover for those patients with higher miR-10b RERs, the identification of agents able to specifically silence miR-10b in cancer cell or modulate its downstream effectors may provide new therapeutic strategies for treating metastatic breast cancer.

## Methods

### Study design

**Figure 2:**
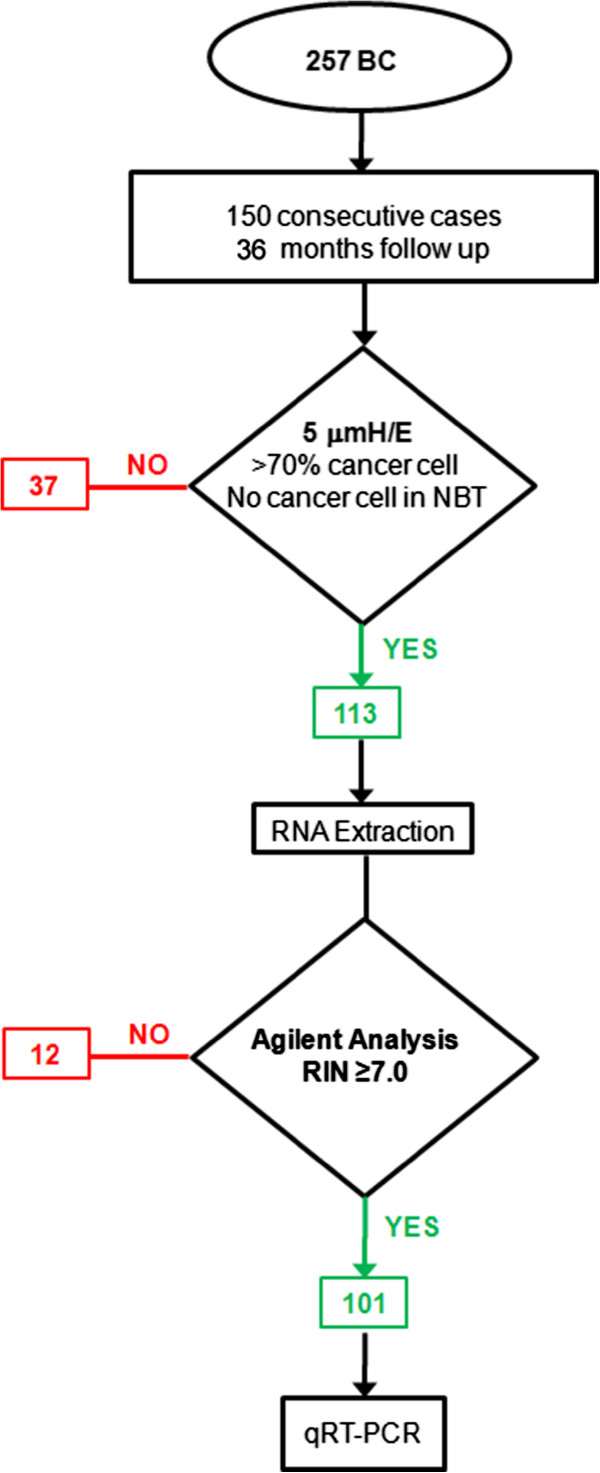
Diagram showing cases selection and RNA quality evaluation.

This study is part of a single institution project initiated in 2006, aimed to the identification of novel biomarkers predicting disease progression and metastases development in breast cancer patients. The study is conducted according to the REporting of tumor MARKer Studies (REMARK) guideline [26] and a prospectively written research, pathologic evaluation, and statistical analysis plan. Paired breast cancer and normal mammary tissues are collected at the Breast-Unit, IRCCS “Casa Sollievo della Sofferenza”. Upon receipt from surgery, tissue from the bulk of the tumor, and normal breast tissue at least 2 cm distant from cancer are sampled by a pathologist (MC), immediately frozen in liquid nitrogen and stored at −80°C until used. For legal reason only one normal and one tumor specimen (approximately 50–100 mg of frozen tissue in weight) can be collected from each patient. Prior written and informed consent is obtained from each patient in accordance with Institutional Guidelines. In order to be included in the study, patients must be female, aged more than 18 years, and tumor must be more than 1.0 cm in diameter due to legal reasons. We selected among the 257 breast cancer cases collected from January 2006 to December 2011, 150 consecutive cases with at least 36 months follow-up (Figure 2). For each case a 5 μm eosin/ematoxylin stained section was prepared to ensure that each tumor sample contained more than 70% of cancer cells and to confirm the absence of tumor cells in the normal specimen. After this analysis, 113 samples were suitable for RNA extraction. Additional 12 cases were excluded because RNA showed a RNA Integrity Number (RIN) <7.0 (n = 101).

### Clinicopathological data

Pathological assessment includes evaluation of histological type, grade and stage. Estrogen Receptor (ER), Progesterone Receptor (PgR), KI-67 labelling index and HER2 expression were evaluated by immunohistochemistry [27,28]. The Nottingham Prognostic Index (NPI) score was calculated according to the following formula: NPI = 0.2xT(cm) + N(1–3) + G(1–3), where T is the maximum diameter in cm, N the number and the level of node metastases (1 = no positive axillary lymph nodes; 2 = 1–3 positive axillary lymph nodes or involvement of a node in the internal mammary chain; 3 = more than three positive axillary lymph nodes or involvement of both axillary and internal mammary lymph nodes) and G the Elston and Ellis grade. Patients are classified at low risk for NPI less or equal to 3.4, at intermediate risk for NPI between 3.4 and 5.4, and at high risk for NPI over 5.4 [29].

### RNA extraction and Reverse Transcription (RT)

According to Trizol reagent protocol (Life Technologies) 80 mg of frozen specimen were carefully and mechanically homogenized and the mixture was transferred into a clean 1.5 ml tube using a sterile scraper. Total RNA was extracted from samples using the TRIzol reagent according to the manufacturer’s instructions. RNA was eluted in RNAse free-water and stored at −80°C until used. RNA quality was measured by using 2100 Expert Analyzer (Agilent Technology) and only RNAs with RNA Integrity Number (RIN) ≥7.0 were processed. RNA concentration was quantified by the absorbance measurement at 260 and 280 nm using the NanoDropTM.1000 spectrophotometer (NanoDrop Technologies).

Single-stranded cDNA was synthesized from 5.5 ng of total RNA using 50 nM specific stem-loop RT primers (miR-10b P/N 4373152 and RNU48 P/N 4373383), 1X RT buffer, dNTPs (each at 0.25 mM), 0,25 U/μl RNase inhibitor and 3.33 U/μl MultiScribe reverse transcriptase. 15 μl reactions were incubated in a GeneAmp PCR System 9700 Thermocycler at 16°C for 30 min, 42°C for 30 min and 85°C for 5 min. RT positive and negative controls were included in each batch of reactions. All reagents were purchased from Life Technologies.

### Quantitative reverse transcription polymerase chain reaction (RT-qPCR) analysis of miR-10b

A relative quantification method with standard curve was developed to determine miR-10b expression in tissues [30]. PCR fragments for the miR-10b and for RNU48 endogenous control were generated using TaqMan miRNA assay (miR-10b P/N 4373152 and RNU48 P/N 4373383, Life Technologies), cloned in the StrataClone™ PCR Cloning Vector pSC-A (Stratagene®) and introduced in StrataClone™SoloPack® Competent Cells. Plasmid DNA from the selected transformant cells was isolated by using the QIAprep® Spin Miniprep Kit (Qiagen) and linearised with Not I (Amersham) Concentration value of plasmid DNA was measured by spectrophotometry and the plasmid copy number was calculated using the following formula: (X μg/μl plasmid DNA/(plasmid and insert length) × 660 g/mole) × 6.023 × 10^23^ = Y molecular number/μl. X represents the concentration of recombinant plasmid DNA, 660 g/molecule the average MW of a double-stranded DNA molecule and Y represents copy number. Five plasmid dilutions of pSC-A_miR-10b and pSC-A_RNU48 (in the range of 1 × 10^6^ copies to 1 × 10^2^ copies) were used to construct the five points calibration curves for real-time PCR.

Real-time PCR reactions were performed in 384-well plates on ABI PRISM 7900HT Sequence Detection System (Life Technologies). 10 μl of reaction mix contained 0.5 μl of TaqMan microRNA assay mix, 5 μl of TaqMan Universal PCR Master Mix, No AmpErase® UNG (Life Technologies) and 1 μl of template. PCR conditions were as follows: at 95°C for 10 min, following by 40 cycles (95°C for 15 s, 60°C for 1 min). Each plate included the miR-10b and RNU48 calibration curves, paired normal and tumour cDNA samples from patients, positive and negative controls of reverse transcription and multiple water blanks; all samples were run in triplicates. The analysis was performed by using SDS 2.4 software (Life Technologie). Standard curves were constructed by plotting the threshold cycle (Ct) values against logarithm10 of the copy number and fitting by linear least square regression. The level of miR-10b expression in each sample was determined as the ratio of the miR-10b copy number to the RNU48 copy number and then multiplied by 1000 for easier tabulation ((miR-10b/RNU48) × 1000).

For each patient, Relative Expression Ratio (RER) was determined as the ratio of miR-10b expression level in the tumor sample to its expression level in the paired normal tissue as previously described [31].

Efficiency of amplification was calculated for each real-time PCR run for both miR-10b and RNU48 as follows: E = (10^(−1/slope)-1) using the slope of the standard curve plots of Ct versus log input of cDNA. The average slope (s) of the standard curves was −3.481 ± 0.160 for miR-10b and −3.510 ± 0.172 for RNU48, indicating efficiencies of 0.941 ± 0.051 and 0.927 ± 0.058, respectively (Additional file 2: Figure S1a).

### Assessment of precision performance of RT-qPCR

One breast cancer case showing high RER and one breast cancer case showing low RER were used to estimate the precision performance of RT-qPCR assay according to the Clinical and Laboratory Standards Institute (CLSI) recommendation. Intra-run variability was assessed by running real-time PCR reactions in triplicate for each paired tumor and normal sample on a 384 well plate. Inter-run variability was assessed by repeating the assay in five independent real-time PCR runs. Intra- and Inter-run variability among RER values were evaluated from the standard deviation and coefficient of variation (CV) (Additional file 2: Figure S1b).

### Immunohistochemical analysis of HOXD10 protein

Representative tumour blocks were sectioned at 3 μm thickness. Immunohistochemical staining was performed by the streptoavidin-biotin method. Endogenous peroxidase in the section was blocked by incubation with 3% hydrogen peroxide. A rabbit polyclonal antibody against the human HOXD10 (H-80: sc-66926; Santa Cruz Biotechnology) was used as primary antibody at a 1/100 dilution. Sections were incubated with LSAB2 (Dakocytomation, Carpinteria, CA). 3-30-diaminobenzidine was used for colour development and hematoxylin was used for counterstaining. Negative controls were obtained by omitting primary antibody.

### Statistical analysis

Patients’ baseline characteristics were reported as median along with Inter Quartile Range (IQR) or frequencies and percentages for continuous and categorical variables, respectively. Time-to-event analysis was performed for patients without metastases at diagnosis by univariable and multivariable proportional hazards Cox regression models. Models included: miR-10b RERs, T, N, Grade, ER, PgR, HER2 and KI67. Risks were reported as Hazard Ratios (HR) along with their 95% Confidence Interval (CI 95%). Overall Survival (OS) was defined as the time between the enrollment date and cancer related death. Disease Free Survival (DFS) was defined as the time between the enrollment date and the tumor progression. Metastasis Free Survival (MFS) was defined as the time between the enrollment date and the development of distant metastases.

The assumption of proportionality of the hazards was tested by using scaled Schoenfeld residuals [32]. For the miR-10b RERs only, HR were reported for each unitary increment of 0.1 expression level (Additional file 3: Table S4).

Predicted risk probabilities were derived from the estimated Cox regression models.

Models’ calibration, i.e. the agreement between observed outcomes and predictions, was assessed using the survival-based Hosmer-Lemeshow (HL) goodness-of-fit test, a chi-squared test based on grouping observations into deciles of predicted risk and testing associations with observed outcomes.

Models’ discrimination, i.e. the ability to distinguish subjects who will develop an event from those who will not, was assessed by computing the modified C-statistic for censored survival data. Comparison between C-statistics was carried out according to Pencina and colleagues [33].

Reclassification improvement for the prediction of the different endpoints offered by mir-10b RER over the NPI was quantified using the survival-based net reclassification index (NRI), following the Kaplan-Meier approach with one-sided bootstrap-based *p*-values [34,35]. Since no established risk cut-offs were available for our high risk population, the continuous NRI (cNRI) was used.

Improvements in model discriminatory power and risk reclassification were assessed at a time horizon of 36 months, since it is well established that the peak hazard for both breast cancer recurrence and development of distant metastases falls within 24–36 months from surgery [36].

A *p*-value <0.05 was considered for statistical significance. All analyses were performed using SAS Release 9.1.3 (SAS Institute).

## Abbreviations

miRNAs: microRNAs; ER: Estrogen Receptor; PR: Progesterone receptor.

## Competing interests

The authors declare that they have no competing interests.

## Authors’ contributions

PP, RB: Substantial contributions to conception and design. RB, BP developed and validated the qRT-PCR assay; AF, MC, FP performed statistical analyses; MC, TB extracted RNA from tissues; GP, DR, AOM developed the HOXD10 IHC assay; VMV, MM collected clinical follow up data; RM, MC collected tissue specimens; PP, RB, BP, MLP, EM Analysis and interpretation of data. PP, RB, BP, MLP, VMF Writing, review of the manuscript. PP, VMF: Study supervision. All authors read and approved the final manuscript.

## Supplementary Material

Additional file 1: Table S1 Comparison between HOXD10 expression determined by IHC and miR10b expression in breast cancer tissues and paired normal specimens. **Table S2.** Associations of clinicopathological characteristics with miR-10b RERs in the whole patients group. **Table S3.** Univariate Cox regression models evaluating the association between clinicopathological variables and: Overall Survival (OS), Time to Progression (TTP) and Metastases Free Survival (MFS) in the patients’ group without metastases at diagnosis (n=90). Click here for file

Additional file 2: Figure S1 Precision of RT-qPCR assay. a) Efficiency of standard curves relative to the 10 plates run in the study for miR-10b and RNU48. b) Intra- and Inter-assay variability of RT-qPCR. Click here for file

Additional file 3: Table S4 Score test of proportional hazards assumption, based on scaled Schoenfeld residuals. Click here for file
