# Supplementary material for: Evaluation of microRNA-10b prognostic significance in a prospective cohort of breast cancer patients
Source: Mol Cancer. 2014 Jun 4;13:142. doi: 10.1186/1476-4598-13-142 (PMC4055397; doi:10.1186/1476-4598-13-142)
Supplement: Additional file 1: Table S1 — Comparison between HOXD10 expression determined by IHC and miR10b expression in breast cancer tissues and paired normal specimens. Table S2. Associations of clinicopathological characteristics with miR-10b RERs in the whole patients group. Table S3. Univariate Cox regression models evaluating the association between clinicopathological variables and: Overall Survival (OS), Time to Progression (TTP) and Metastases Free Survival (MFS) in the patients’ group without metastases at diagnosis (n=90). [file 1476-4598-13-142-S1.doc]

**Table S1.** Comparison between HOXD10 expression determined by IHC and miR10b expression in breast cancer tissues and paired normal specimens

| **Sample code*** | **HOXD10** | **miR10b** |
| --- | --- | --- |
|  | **% positive cells** | **expression** |
| **BC1** | 25 | 289,27 |
| **BC2** | 50 | 136,49 |
| **BC3** | 50 | 44,02 |
| **BC4** | 20 | 37,11 |
| **BC5** | 70 | 14,84 |
| **BC6** | 100 | 16,60 |
| **BC7** | 70 | 12,83 |
| **BC8** | 100 | 16,18 |
| **BC9** | 40 | 20,63 |
| **BC10** | 70 | 126,26 |
| **NBT1** | 30 | 444,66 |
| **NBT2** | 0 | 965,67 |
| **NBT3** | 50 | 62,71 |
| **NBT4** | 50 | 47,51 |
| **NBT5** | 0 | 817,81 |
| **NBT6** | 50 | 2478,88 |
| **NBT7** | 60 | 30,38 |
| **NBT8** | 0 | 330,66 |
| **NBT9** | 10 | 207,45 |
| **NBT10** | 50 | 174,40 |
| **HSB1** | 100 |  |
| **HSB2** | 70 |  |
| **HSB3** | 70 |  |

*BC, breast cancer tissue, NBT normal breast tissue, HSB, breast tissue from healthy individuals

**Table S2.** Associations of clinicopathological characteristics with miR-10b RERs in the whole patients group.

| **Characteristics** | **N°** | **miR-10b**  **RERs** | ***p*-value** |
| --- | --- | --- | --- |
|
| **Age** | 101 | -0.01 | 0.910 |
| **Tumor size** | 99 | -0.03 | 0.769 |
| **Histotype** |  |  |  |
| Ductal | 92 | 0.10 (0.05-0.32) | 0.77 |
| Lobular | 7 | 0.10 (0.01-0.27) |
| Others | 3 | 0.26 (0.03-0.49) |
| **Tumor** |  |  |  |
| T1c | 27 | 0.08 (0.03-0.19) | 0.19 |
| T2 | 45 | 0.11 (0.06-0.34) |
| T3 | 4 | 0.06 (0.03-0.89) |
| T4 | 25 | 0.20 (0.05-0.57) |
| **Lymph nodes** |  |  |  |
| N0 | 34 | 0.09 (0.05-0.34) | 0.56 |
| N1-N2-N3 | 67 | 0.13 (0.03-0.32) |
| **Metastases** |  |  |  |
| M0 | 90 | 0.09 (0.04-0.29) | **0.028** |
| M1 | 11 | 0.25 (0.11-1.02) |
| **Stage** |  |  |  |
| I | 15 | 0.07 (0.04-0.19) | 0.13* |
| II | 44 | 0.09 (0.05-0.30) |
| III | 31 | 0.09 (0.03-0.49) |
| IV | 11 | 0.25 (0.10-1.01) |
| **ER** |  |  |  |
| Negative | 38 | 0.10 (0.05-0.31) | 0.96 |
| Positive | 63 | 0.11 (0.04-0.32) |
| **PgR** |  |  |  |
| Negative | 50 | 0.11 (0.05-0.40) | 0.47 |
| Positive | 51 | 0.10 (0.04-0.27) |
| **Receptor Classification** |  |  |  |
| Hormone Receptor Positive | 63 | 0.11 (0.04-0.32) | 0.69 |
| Triple Negative | 20 | 0.08 (0.05-0.21) |
| Her2/neu amplified only | 18 | 0.15 (0.04-0.53) |
| **Grade** |  |  |  |
| G1 | 11 | 0.23 (0.05-0.43) | 0.19 |
| G2 | 38 | 0.10 (0.05-0.32) |
| G3 | 40 | 0.08 (0.04-0.28) |
| **HER2 amplification/overexpression** | | | |
| Negative | 66 | 0.10 (0.05-0.26) | 0.85 |
| Positive | 30 | 0.15 (0.04-0.37) |
| **KI67 labeling index** |  |  |  |
| <30% | 46 | 0.09 (0.04-0.32) | 0.56 |
| >30% | 44 | 0.10 (0.05-0.42) |
| **First Metastatic site** |  |  |  |
| Brain | 6 | 0.47 (0.20-1.62) | **0.03** |
| Bone | 19 | 0.09 (0.014-0.58) |
| Lung | 11 | 0.09 (0.06-0.70) |  |
| Other sites | 5 | 0.19 (0.03-0.49) |  |
| **NPI^#^** | | | |
| Low Risk | 16 | 0.18 (0.07-0.41) | 0.47 |
| Intermediate Risk | 43 | 0.09 (0.05-0.27) |
| High Risk | 30 | 0.10 (0.02-0.57) |

Abbreviations: ER, Estrogen Receptor; PgR, Progesterone Receptor.

miR-10b RERs for categorical variables are reported as medians along with lower and upper quartiles (range). Differences between groups were assessed by two-sample *t test* or one-way ANOVA and for stage and grade by test for linear trend from ANOVA model using log-transformed miR-10b RERs. Pearson correlations were estimated to assess relationship between miR-10b RERs and continuous variables.*Brain versus bone metastases; **^#^**NPI was calculated for patients without metastases at diagnosis.

**Table S3.** Univariate Cox regression models evaluating the association between clinicopathological variables and: Overall Survival (OS), Time to Progression (TTP) and Metastases Free Survival (MFS) in the patients’ group without metastases at diagnosis (n=90).

1. **Overall Survival (OS)**

| **Variable** | **Ev/tot** | **Category** | **HR** | **95%CI** | ***p*-value** |
| --- | --- | --- | --- | --- | --- |
| Tumor | 18/90 | T4 vs T1 | 8.36 | 1.81-38.76 | **0.007** |
|  |  | T3 vs T1 | 5.82 | 0.53-64.45 | 0.151 |
|  |  | T2 vs T1 | 2.09 | 0.42-10.35 | 0.369 |
| Lymph node | 18/90 | Positive vs Negative | 4.66 | 1.07-20.29 | **0.040** |
| Grading | 15/89 | G3 vs G1 | 3.66 | 0.47-28.58 | 0.215 |
|  |  | G2 vs G1 | 0.84 | 0.09- 8.10 | 0.882 |
| Stage | 18/90 | III vs I | 6.32 | 0.82-48.67 | 0.077 |
|  |  | II vs I | 1.67 | 0.19-14.34 | 0.638 |
| ER status | 18/90 | Positive vs negative | 0.25 | 0.09-0.68 | **0.007** |
| PgR status | 18/90 | Positive vs Negative | 0.32 | 0.11-0.90 | **0.031** |
| HER2 | 18/87 | Positive vs Negative | 0.84 | 0.30-2.34 | 0.733 |
| KI67 | 16/80 | <30% vs >30% | 0.51 | 0.19-1.41 | 0.196 |
| Receptor Classification | 18/90 | Her2/neu amplified only vs Triple Negative | 0.30 | 0.08-1.11 | 0.071 |
|  |  | Hormone Receptor positive vs Triple Negative | 0.16 | 0.05-0.45 | **<0.001** |
| NPI | 15/89 | High risk vs Low risk | 11.68 | 1.51-90.10 | **0.018** |
|  |  | Intermediate risk vs Low risk | 0.83 | 0.07-9.12 | 0.877 |

1. **Disease Free Survival (DFS)**

| **Variable** | **Ev/tot** | **Category** | **HR** | **95%CI** | ***p*-value** |
| --- | --- | --- | --- | --- | --- |
| Tumor | 34/90 | T4 vs T1 | 5.76 | 2.02-16.42 | **0.001** |
|  |  | T3 vs T1 | 6.08 | 1.18-31.43 | **0.031** |
|  |  | T2 vs T1 | 2.17 | 0.79-5.97 | 0.134 |
| Lymph node | 34/90 | Positive vs Negative | 2.62 | 1.14-6.02 | **0.023** |
| Grade | 29/89 | G3 vs G1 | 2.06 | 0.61-6.94 | 0.245 |
|  |  | G2 vs G1 | 0.51 | 0.13-2.02 | 0.335 |
| Stage | 18/90 | III vs I | 14.59 | 1.96-108.85 | **0.009** |
|  |  | II vs I | 4.59 | 0.60-35.07 | 0.142 |
| ER status | 34/90 | Positive vs negative | 0.46 | 0.23-0.92 | **0.027** |
| PgR status | 34/90 | Positive vs Negative | 0.60 | 0.30-1.20 | 0.151 |
| HER2 | 34/87 | Positive vs Negative | 1.57 | 0.79-3.15 | 0.200 |
| KI67 | 31/80 | <30% vs >30% | 0.62 | 0.31-1.27 | 0.196 |
| Receptor Classification | 34/90 | Her2/neu amplified only vs Triple Negative | 0.67 | 0.26-1.73 | 0.406 |
|  |  | Hormone Receptor positive vs Triple Negative | 0.39 | 0.18-0.84 | **0.017** |
| NPI | 29/89 | High risk vs Low risk | 10.57 | 2.43-45.99 | **0.002** |
|  |  | Intermediate risk vs Low risk | 2.05 | 0.45-9.38 | 0.353 |

1. Metastases Free Survival (MFS)

| **Variable** | **Ev/tot** | **Category** | **HR** | **95%CI** | ***p*-value** |
| --- | --- | --- | --- | --- | --- |
| Tumor | 30/90 | T4 vs T1 | 6.16 | 1.95-19.44 | **0.002** |
|  |  | T3 vs T1 | 7.87 | 1.44-43.14 | **0.017** |
|  |  | T2 vs T1 | 2.38 | 0.77-7.32 | 0.130 |
| Lymph node | 30/90 | Positive vs Negative | 2.60 | 1.06-6.36 | **0.037** |
| Grade | 26/89 | G3 vs G1 | 3.05 | 0.71-13.14 | 0.134 |
|  |  | G2 vs G1 | 0.65 | 0.13-3.35 | 0.607 |
| Stage | 26/90 | III vs I | 13.23 | 1.77-98.93 | **0.012** |
|  |  | II vs I | 3.48 | 0.44-27.17 | 0.235 |
| ER status | 30/90 | Positive vs negative | 0.44 | 0.21-0.92 | **0.029** |
| PgR status | 30/90 | Positive vs Negative | 0.56 | 0.27-1.16 | 0.119 |
| HER2 | 30/87 | Positive vs Negative | 1.67 | 0.80-3.47 | 0.172 |
| KI67 | 27/80 | <30% vs >30% | 0.49 | 0.22-1.07 | 0.072 |
| Receptor Classification | 30/90 | Her2/neu amplified only vs Triple Negative | 0.61 | 0.22-1.69 | 0.345 |
|  |  | Hormone Receptor positive vs Triple Negative | 0.36 | 0.16-0.82 | **0.015** |
| NPI | 26/89 | High risk vs Low risk | 9.94 | 2.29-43.25 | **0.002** |
|  |  | Intermediate risk vs Low risk | 1.41 | 0.29-6.79 | 0.668 |

Abbreviations: ER, estrogen receptor; PgR Progesterone Receptor; HR, Hazard Ratio; 95%CI, 95% Confidence Interval

HR greater 1.0 means a higher risk to develop the event, HR below 1.0 means a lower risk to develop the event

**Table S4.** Association of miR-10b RERs and survival in the subgroup of patients treated with Hormone Therapy and Chemotherapy (HT+CT) or Chemotherapy with or without association with anti-HER2 treatments (CT).

| **Variable** |  | **Ev/Tot** | **HR** | **95%CI** | ***p-*value** |
| --- | --- | --- | --- | --- | --- |
| **Metastases Free Survival (MFS**) | | | | | |
| HT+CT | | 13/53 | 7.21 | 1.11-46.89 | 0.039 |
| CT | | 17/34 | 4.71 | 0.49-45.11 | 0.179 |

| **Disease Free Survival (DFS)** | | | | |
| --- | --- | --- | --- | --- |
| HT+CT | 15/53 | 5.39 | 0.91-31.85 | 0.063 |
| CT | 17/34 | 4.59 | 0.57-36.70 | 0.151 |
