# Supplementary figures and images for: Evaluation of microRNA-10b prognostic significance in a prospective cohort of breast cancer patients
Source: Mol Cancer. 2014 Jun 4;13:142. doi: 10.1186/1476-4598-13-142 (PMC4055397; doi:10.1186/1476-4598-13-142)

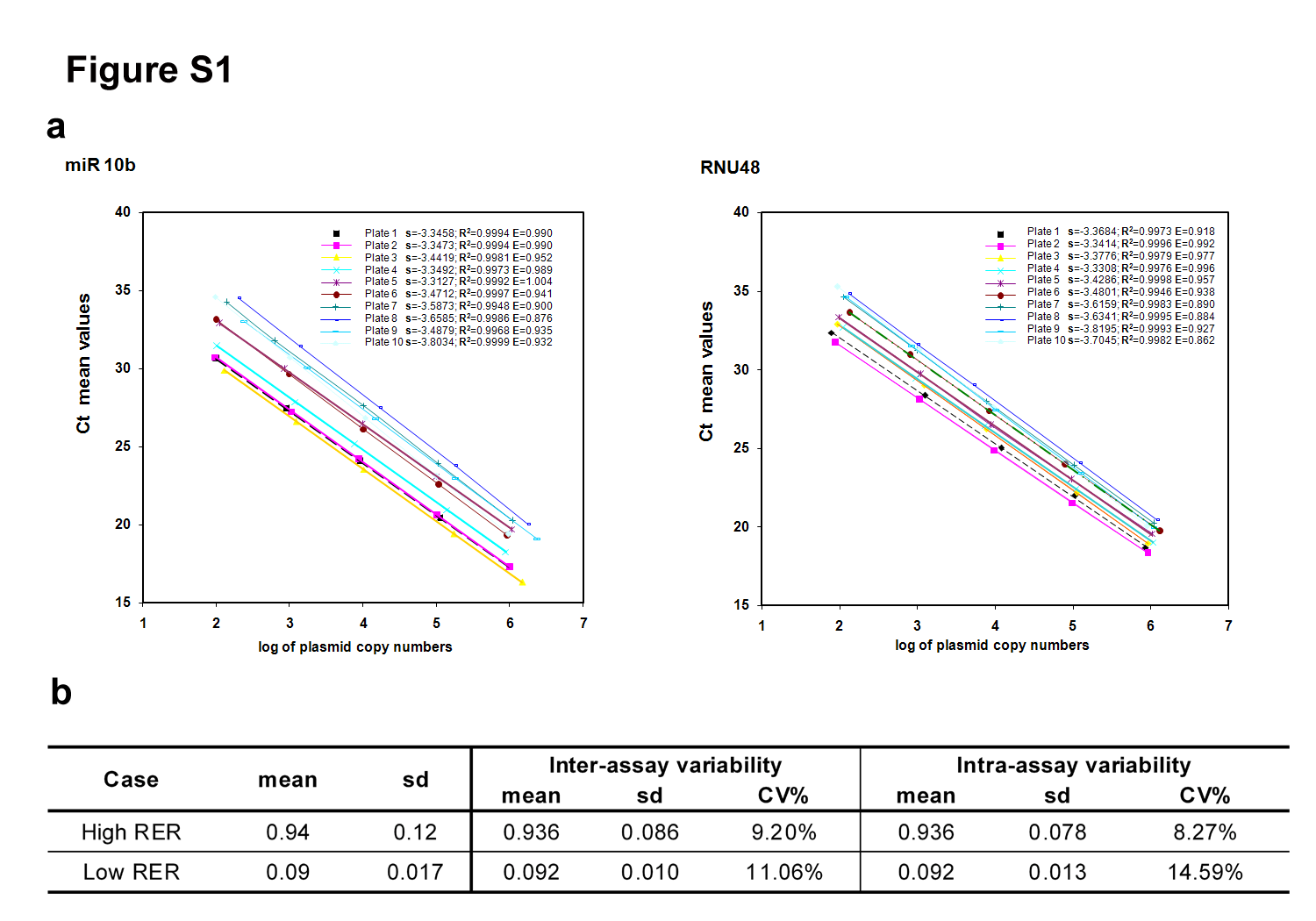

Supplement: Additional file 2: Figure S1 — Precision of RT-qPCR assay. a) Efficiency of standard curves relative to the 10 plates run in the study for miR-10b and RNU48. b) Intra- and Inter-assay variability of RT-qPCR. [file 1476-4598-13-142-S2.tiff]
