# Supplementary material for: Evaluation of microRNA-10b prognostic significance in a prospective cohort of breast cancer patients
Source: Mol Cancer. 2014 Jun 4;13:142. doi: 10.1186/1476-4598-13-142 (PMC4055397; doi:10.1186/1476-4598-13-142)
Supplement: Additional file 3: Table S4 — Score test of proportional hazards assumption, based on scaled Schoenfeld residuals. [file 1476-4598-13-142-S3.docx]

**Table for peer Reviewer.** Score test of proportional hazards assumption, based on scaled Schoenfeld residuals

| **Outcome** | **Model** | **Variables** | **Category** | **Rho*** | **Chi-square** | **df** | **p-value** |
| --- | --- | --- | --- | --- | --- | --- | --- |
| Overall Survival (OS) | Univariate | miR-10b RERs | --- | -0.071 | 0.107 | 1 | 0.743 |
|  | Multivariate | miR-10b RERs | --- | -0.219 | 1.565 | 1 | 0.211 |
|  |  | Tumor | T2 vs. T1 | 0.051 | 0.067 | 1 | 0.796 |
|  |  |  | T3 vs. T1 | -0.361 | 2.727 | 1 | 0.099 |
|  |  |  | T4 vs. T1 | -0.223 | 0.974 | 1 | 0.324 |
|  |  | Lymph node | Positive vs. Negative | -0.401 | 1.275 | 1 | 0.259 |
|  |  | ER status | Positive vs. Negative | 0.033 | 0.027 | 1 | 0.870 |
|  |  | PgR status | Positive vs. Negative | 0.084 | 0.115 | 1 | 0.734 |
|  |  | KI67 | --- | -0.140 | 0.380 | 1 | 0.538 |
|  |  | HER2 | Positive vs. Negative | 0.325 | 5.180 | 1 | 0.023 |
|  |  | Grade | G2 vs. G1 | -0.164 | 0.327 | 1 | 0.567 |
|  |  | Grade | G3 vs. G1 | -0.103 | 0.165 | 1 | 0.685 |
|  |  | Global test | --- | --- | 10.921 | 11 | 0.450 |
| Disease Free Survival (DFS) | Univariate | miR-10b RERs | --- | -0.262 | 3.031 | 1 | 0.082 |
|  | Multivariate | miR-10b RERs | --- | -0.083 | 0.301 | 1 | 0.583 |
|  |  | Tumor | T2 vs. T1 | -0.094 | 0.345 | 1 | 0.557 |
|  |  |  | T3 vs. T1 | -0.244 | 2.764 | 1 | 0.096 |
|  |  |  | T4 vs. T1 | -0.093 | 0.320 | 1 | 0.572 |
|  |  | Lymph node | Positive vs. Negative | -0.074 | 0.172 | 1 | 0.678 |
|  |  | ER status | Positive vs. Negative | 0.017 | 0.013 | 1 | 0.908 |
|  |  | PgR status | Positive vs. Negative | 0.077 | 0.223 | 1 | 0.636 |
|  |  | KI67 | --- | 0.071 | 0.259 | 1 | 0.611 |
|  |  | HER2 | Positive vs. Negative | 0.176 | 1.532 | 1 | 0.216 |
|  |  | Grade | G2 vs. G1 | 0.079 | 0.238 | 1 | 0.626 |
|  |  | Grade | G3 vs. G1 | -0.020 | 0.012 | 1 | 0.913 |
|  |  | Global test | --- | --- | 5.276 | 11 | 0.917 |
| Metastasis-free survival (MFS) | Univariate | miR-10b RERs | --- | -0.246 | 2.591 | 1 | 0.107 |
|  | Multivariate | miR-10b RERs | --- | -0.045 | 0.091 | 1 | 0.763 |
|  |  | Tumor | T2 vs. T1 | -0.083 | 0.250 | 1 | 0.617 |
|  |  |  | T3 vs. T1 | -0.182 | 1.309 | 1 | 0.253 |
|  |  |  | T4 vs. T1 | -0.057 | 0.121 | 1 | 0.728 |
|  |  | Lymph node | Positive vs. Negative | 0.003 | 0.000 | 1 | 0.988 |
|  |  | ER status | Positive vs. Negative | 0.050 | 0.110 | 1 | 0.740 |
|  |  | PgR status | Positive vs. Negative | 0.111 | 0.408 | 1 | 0.523 |
|  |  | KI67 | --- | 0.159 | 1.095 | 1 | 0.295 |
|  |  | HER2 | Positive vs. Negative | 0.147 | 0.934 | 1 | 0.334 |
|  |  | Grade | G2 vs. G1 | -0.021 | 0.012 | 1 | 0.912 |
|  |  | Grade | G3 vs. G1 | -0.095 | 0.216 | 1 | 0.642 |
|  |  | Global test | --- | --- | 5.375 | 11 | 0.912 |

*Rho is the correlation coefficient of the scaled Schoenfeld residuals with the follow-up time variable
